# Circulating β-endorphin, adrenocorticotrophic hormone and cortisol levels of stallions before and after short road transport: stress effect of different distances

**DOI:** 10.1186/1751-0147-50-6

**Published:** 2008-03-03

**Authors:** Esterina Fazio, Pietro Medica, Vincenzo Aronica, Loredana Grasso, Adriana Ferlazzo

**Affiliations:** 1 Department of Morphology, Biochemistry, Physiology and Animal Production, Unit of Veterinary Physiology, Faculty of Veterinary Medicine, University of Messina, 98168 Messina, Italy; 2 Unit of Clinical Biochemistry, University General Hospital (Gaetano Martino), University of Messina, 98168 Messina, Italy

## Abstract

**Background:**

Since transport evokes physiological adjustments that include endocrine responses, the objective of this study was to examine the responses of circulating β-endorphin, adrenocorticotrophic hormone (ACTH) and cortisol levels to transport stress in stallions.

**Methods:**

Forty-two healthy Thoroughbred and crossbred stallions were studied before and after road transport over distances of 100, 200 and 300 km. Blood samples were collected from the jugular vein: first in a single box immediately before loading (pre-samples), then immediately after transport and unloading on arrival at the breeding stations (post-samples).

**Results:**

An increase in circulating β-endorphin levels after transport of 100 km (*P *< 0.01), compared to basal values was observed. Circulating ACTH levels showed significant increases after transport of 100 km (*P *< 0.001) and 200 km (*P *< 0.001). Circulating cortisol levels showed significant increases after road transport over distances of 100, 200 and 300 km (*P *< 0.001). An effect of transport on β-endorphin, ACTH and cortisol variations was therefore evident for the different distances studied. No significant differences (*P *> 0.05) between horses of different ages and different breeds were observed for β-endorphin, ACTH and cortisol levels.

**Conclusion:**

The results obtained for short term transportation of stallions showed a very strong reaction of the adrenocortical system. The lack of response of β-endorphin after transport of 200–300 km and of ACTH after transport of 300 km seems to suggest a soothing effect of negative feedback of ACTH and cortisol levels.

## Background

Competitions, breeding, leisure activities, sale or slaughter are the most usual reasons for transporting horses. The necessity of transporting live animals has increased the need to better evaluate horse welfare and health, and thus to verify the effects of transport stress on the variables related to physiological adaptations. Studies to determine the amount of stress experienced by horses during transport have yielded widely varying results. Results are difficult to interpret because transportation involves a range of potential stressors, such as loading, unloading, confinement, vibration, changes in temperature and humidity, inadequate ventilation, space allowed [1] and, frequently, deprivation of food and water. Recently, air stables have proven to be a convenient way of transporting horses on international flights, and caused no discernible ill effects on the horses studied [2]. The effects of long distance transport stress have been widely reported and considered in relation to behavioural [3-5], functional [6-10], endocrine and biochemical variables [11,12], and also in terms of the impact on the immune system [13-15]. The effects of transportation have also been studied with regard to performance [16,17] and reproduction [18,19]. In general, transport by road is more uncomfortable for animals than by rail or air. Moreover, there is ample evidence demonstrating that long periods of road transport have a greater impact on welfare than shorter transport carried out in the same conditions, because of the obvious influence of the prolonged time and the presence of a number of stressors [10,20]. During transport, horses are forced to maintain unnatural body postures for long periods. If this is combined with the additional stress of being placed in an unfamiliar environment, it is likely to have a detrimental effect on the welfare, and even the performance, of some horses [5].

In the case of short-distance transport of horses, however, most endocrine responses have not been extensively studied. In fact, it has been shown that an increased incidence of disease occurs with increased transport distance or travelling time, and that restricting travel time to less than 12 hours may greatly reduce the probability of a horse experiencing transported-related pyrexia or respiratory disease [21].

There is little information available regarding the physiological responses of horses to one to three hours of transportation using a commercial trailer during springtime.

In light of this, the aim of this study was to evaluate the response of β-endorphin, adrenocorticotrophic hormone (ACTH) and cortisol before and after short road transport to breeding stations, with distances ranging between 100–300 km.

## Methods

### Animals

The study was carried out on a total of 42 healthy Thoroughbred and crossbred stallions, ranging in age from 4 to 20 years and weighing 530 ± 20 kg. The horses were transported from their previous stabling to various breeding stations. All horses had previous trailing experience.

All methods and the procedures used in this study were reviewed and approved by the Messina University Institutional Board for the Care and Use of Animals.

### Experimental design

Preliminary procedures (handling, loading, confinement and unloading) were undertaken by the same staff and blood sampling was always carried out by the same operator. All the journeys took place during the months of March and April. Environmental temperature and relative humidity were 19°C and 62%, respectively. Temperature and relative humidity inside the trailers during transport were 22°C and 80% after 1 h, 23°C and 81% after 2 h, and 22°C and 65% after 3 h. These were continuously monitored using a Hygrothermograph ST-50 (Sekonic Corporation, Tokio, Japan), placed near the center of the trailer. The commercial trailer used was 9.5 m long and 2.5 m wide with a ceiling height of 2.5 m. Six single compartments with swinging gates were available (6 horses per load). Stocking density was about 2 m^2^/horse. Rubber padding lined the sides of the trailers from the floor up to an approximate height of 1.2 m. The number of horses per load, the floor area available, distance travelled, and time between loading and unloading were recorded. Feed and water were provided before loading but not during transportation. The horses were usually fed twice a day (at 07.00 a.m. and 07.00 p.m.) with hay (2 kg), bran (1 kg) and concentrate (broad bean, barley, maize, carob) (4 kg) and were given water *ad libitum*. The stallions were transported by road in a commercial trailer for a period of 1–3 h depending on distance. They were divided into 3 different groups, on the basis of the road transport distances: Group I: 100 km; Group II: 200 km; Group III: 300 km.

### Processing of samples and analytical methods

Blood samples were collected from the jugular vein. This procedure took just a few seconds for each horse and physical restraint was needed; this was achieved by haltering each horse. The samples were collected immediately before loading, while horses were in a single box, at 8.00 a.m. (pre-samples) and immediately after transport and unloading, on arrival at the breeding stations (post-samples): at 9.00 a.m. for Group I, 10.00 a.m. for Group II and 11.00 for Group III.

Blood samples were collected using evacuated tubes (Venoject, Terumo^®^; Belgium) and were transferred into a polypropylene tube containing EDTA (1 mg/ml of blood) and aprotinin (500 KIU/ml of blood, ICN Biomedicals Inc., Aurora, Ohio) kept at 4°C. Plasma samples were harvested after centrifugation at 3,000 g for 15 min at 4°C and stored at -80°C until analysed.

Peptides were extracted from plasma samples using 1% trifluoroacetic acid (TFA, HPLC grade) and by elution with 60% acetonitrile (HPLC Grade) in 1% TFA.

Plasma β-endorphin concentrations were measured in duplicate utilizing a commercial RIA kit (Peninsula Lab., Inc., Belmont, CA, USA) for human β-endorphin, with 100% cross-reactivity with equine β-endorphin [22-24]. The hormone assay utilised had a detection range for β-endorphin of 3–371 pmol/l. Intra- and interassay coefficient of variation (CV) were 7% and 15%, respectively.

Serum ACTH concentrations were analysed in duplicate using a commercially available radioimmunoassay kit (ELSA-ACTH, CIS-BioInternational, Gif-sur-Yvette, France) suitable for equine use [25]. The hormone assay utilised had a ACTH detection range of 0–440 pmol/l. Intra- and interassay CV were 15% and 6%, respectively.

Serum cortisol concentrations were analysed in duplicate using a commercially available immunoenzymatic kit (Roche Diagnostics GmbH, Mannheim, Germany). The hormone assay utilised had a cortisol detection range of 0–1380 nmol/l. Intra- and interassay CV were 4.6 % and 6.9%, respectively.

### Statistics

Data are presented as mean ± standard deviation (SD). A one way repeated measures analysis of variance (RM-ANOVA) was applied to determine whether transport stress had any effect on hormonal variations. A paired t-test was used to compare post-transport and basal values within each of the three groups, while an unpaired t-test was used to compare basal values between the three groups. The level of significance was set at *P *< 0.05. All calculations were performed using the PRISM package (GraphPad Software Inc., San Diego, CA, USA).

## Results

**Figure 1:**
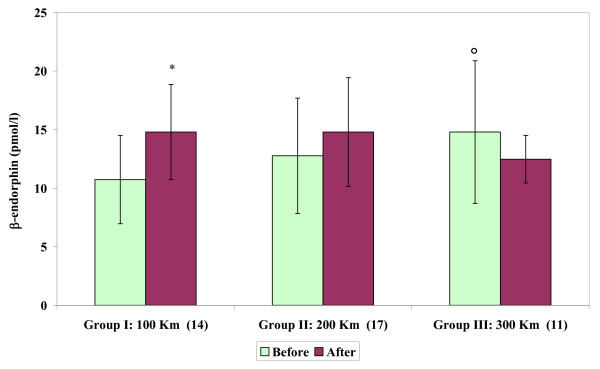
**Circulating β-endorphin concentrations (mean ± SD) of stallions before and after short road transport of different distances**. Label on X-axis: Groups, Distances (km), number of subjects. Asterisk indicates significant (*P < 0.001) differences vs before. Symbol indicates significant (°*P *< 0.01) differences vs Group I.

Circulating β-endorphin levels showed an increase (Figure 1) after road transport in Group I (100 Km: *P *< 0.01), compared to basal values. Thus, an effect of transport was shown for a distance of 100 km (*P *< 0.001).

Basal β-endorphin levels in Group III were significantly higher (*P *< 0.01) than basal values observed in Group I. No significant differences in basal values of β-endorphin were observed between Groups I and II.

**Figure 2:**
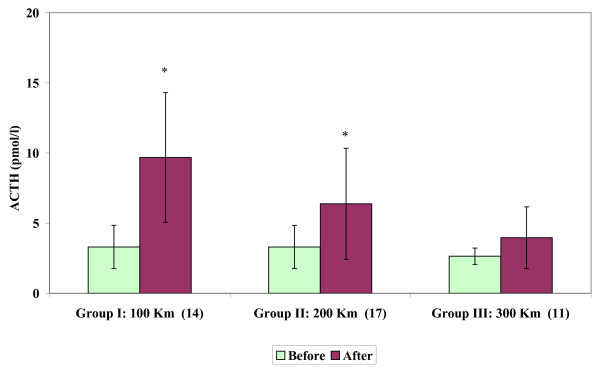
**Circulating ACTH concentrations (mean ± SD) of stallions before and after short road transport of different distances**. Label on X-axis: Groups, Distances (km), number of subjects. Asterisk indicates significant (*P < 0.001) differences vs before.

Circulating ACTH levels (Figure 2) showed increases after transport in Group I (100 km: *P *< 0.001) and in Group II (200 km: *P *< 0.001). Thus, an effect of transport was shown for distances of 100 km (*P *< 0.001) and 200 km (*P *< 0.002).

**Figure 3:**
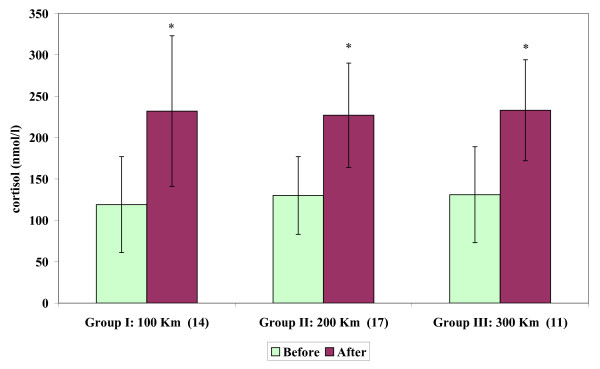
**Circulating cortisol concentrations (mean ± SD) of stallions before and after short road transport of different distances**. Label on X-axis: Groups, Distances (km), number of subjects. Asterisk indicates significant (*P < 0.001) differences vs before.

Circulating cortisol levels (Figure 3) showed significant increases in Groups I, II and III over all the transport distances: 100 km (*P *< 0.001), 200 km (*P *< 0.001) and 300 km (*P *< 0.001).

Significant transport effects were shown for circulating cortisol (*P *< 0.01) levels for all three distances. No significant differences of β-endorphin, ACTH and cortisol levels were observed between young (15 horses, 4 years old) and mature (27 horses, 7–20 years old) stallions, nor between Thoroughbreds and crossbreds, in both basal conditions as well as after transport, regardless of transport distance (100–300 km).

Temperature inside the trailer during transport increased after 2 h (+6%; *P *< 0.05) and decreased after 3 h (-4; *P *< 0.05). Relative humidity decreased only after 3 h (-20; *P *< 0.01).

## Discussion

Many laboratories have established reliable reference values for β-endorphin, ACTH and cortisol values in the blood of healthy horses. Many factors, both endogenous and exogenous, affect hormone secretion and may lead to the misinterpretation of test results when values for individual animals are compared with reference values. In addition, slight variations could be ascribed to differences in techniques and some differences may also be explained by physical and psychological factors. The comparisons of results obtained in this study with published data reported for horses did not reveal any large discrepancies for circulating β-endorphin (8–26 pmol/l) [23,26-30], ACTH (3–7 pmol/l) [31,32] and cortisol (83–359 nmol/l) [2,31,33-35] levels. Any slight variation could be ascribed to differences in techniques.

The results obtained document how the endogenous opioid peptides and the hypophysis-adrenocortical response actively modulate the adaptation to transport stress conditions in horses, albeit in a temporally differentiated way.

Our results confirm data previously obtained in horses, which showed the effects of road transport stress on circulating β-endorphin, ACTH and cortisol levels [11,36,37].

Opioids are involved in many responses to stress [6,38] and regulate various endocrine systems, including the hypothalamic-pituitary-adrenocortical (HPA) axis.

In our experimental conditions, the endogenous opioid system modulated the response to stress, probably more during the earlier phase of transportation (100 km), than during the subsequent phases (for distances of 200 and 300 km). The present study is in line with previous studies [6,22,39] demonstrating that β-endorphin levels immediately increase after the application of a stressor, as in the case of the preliminary phases of short road transport. The decrease in β-endorphin levels detected after road transport of 200–300 km might be explained by a lasting negative feedback effect. Indeed, Li and Chen [40] reported that transportation by road significantly increased plasma concentrations of beta-endorphin-like material (β-END-L1) from a basal value within 30 min; these concentrations were maintained at 45 min and began to decline after 60 min of transport.

The differences observed in basal values of β-endorphin between Groups I and III confirm that endogenous opioid peptides show great individual variation in horses [6].

Our findings suggest that the animals' responses to transport stress are influenced by the different distances and/or duration. However, they do not exclude that individual variations may play a significant role.

In contrast, transportation of acclimatized adult horses for 1 h in a trailer [16,41] or in an enclosed container during flights of 12 to 24 h duration [2] did not result in any change in β-endorphin levels.

Increases in ACTH levels after transport over distances of 100 and 200 km confirm that ACTH must be recognized as an important effector hormone in mediating endocrine responses under conditions of physical or psychological stress [42].

Moreover, concomitant variations in β-endorphin and ACTH levels in response to transport of 100 km confirm concurrent regulation from the intermediate lobe with a substantial release of both hormones from the anterior pituitary gland [43]. In addition, confinement in a vehicle has been shown to cause a significant increase in β-endorphin and ACTH concentrations [40]. This finding confirms that confinement and loading affect β-endorphin and ACTH release, as seen in stallions during the preliminary phases of transport.

Increases in cortisol levels after journeys of 100–300 km confirm that cortisol levels are an indicator of stress in horses [11,12,14,44-50].

Our data showed that cortisol may be useful as an indicator of short-term stress. It must be remembered that cortisol is a time-dependent measure that takes 10 to 20 min to reach peak values [51]. The ability of the adrenocortical gland to produce cortisol, however, continued during transportation and did not decrease with experience when horses underwent short road transport.

Persistent increases in cortisol levels showed no differences relating to the different distances and durations of transport, possibly because of its short half-life of 1 to 1.5 h in horses [52].

We concluded that transport stress provoked the greatest cortisol response to ACTH, which suggested that the transported stallions had continuously used their emergency adrenocortical response regardless of distance and duration. It is well known that cortisol concentrations in resting horses exhibit a daily circadian rhythm [53,54]. However, this factor did not affect the basal cortisol values (pre-sampling) nor the post-transport values (post-sampling) because the percentage increases in cortisol were equal for all three groups and because there were no significant differences between the basal values of the different groups. In addition, it is well known that placing horses in a novel environment obliterates the circadian rhythm in total cortisol concentrations by elevating levels during the time of the normal trough [55].

However, the large incremental rise in cortisol concentration after transport of 100–300 km may be influenced by pituitary activity, exhibited by an increase in ACTH concentrations. Moreover, the positive feedback of ACTH concentrations on cortisol release seemed to change in relation to road transport distances. In fact, the increase of ACTH concentrations progressively decreased after transport as distance increased, and these changes were not concomitant with those of cortisol levels. Furthermore, elevated β-endorphin concentrations after transport may contribute to the release of ACTH hormone, but were limited to the 1 h transport period (100 km). In any case, the release of β-endorphin and ACTH from the pituitary gland can be used as a reliable indication of stress [21,22].

Short transport of 1–3 h could also be an expression of psychological stress, which is usually quantified in terms of ACTH, cortisol, and/or beta-endorphin responses, rather than of physical stress, which can reflect trauma and/or disease, as reported by Leadon [16]. The changes in temperature and relative humidity during transport might have an additional effect on the endocrine responses. Moreover, the wide range of circulating β-endorphin levels recorded for the horses might partly be due to individual differences, as reported in a previous study [6].

However, it can not be excluded that confinement procedures and the stress of novelty, which begin on departure and are maintained throughout the journey, could play a determining role in greater activation of the opioid system and hypophysis-adrenocortical axis response.

In addition, the presence of conspecifics, did not reduce the response to transport stress in stallions already accustomed to transport.

Short distance and duration of transport seemed to greatly modify the stress response, whilst age, breed and experience of horses did not appear to influence it.

## Conclusion

Transport conditions and handling of horses induced significant alterations in common physiological measures of stress, i.e. β-endorphin, ACTH and cortisol concentrations. Transportation of horses induced a very strong reaction of the adrenocortical system, attested during the preliminary phases by both β-endorphin and ACTH increases. Alleviating these stresses in transported animals should therefore be a prime concern for horse welfare and health. Transport is inevitably associated with a stress response but this can be avoided by adequate handling and management. Therefore, the use of hormonal stress markers merits consideration.

As β-endorphin, ACTH and cortisol evaluation in these conditions have been shown to be efficacious in evaluating transport stress in horses they may offer an additional tool by which to do this.

## Competing interests

The author(s) declare that they have no competing interests.

## Authors' contributions

EF was responsible for the study design, preparation and revision of the manuscript. PM was responsible for hormones and statistical analyses. VA carried out the blood sampling. LG was responsible for hormones analyses. AF was responsible for study design and manuscript preparation. All authors read and approved the final manuscript.
